# Side- and Sinus-Specific Relationships between Chronic Rhinosinusitis and Ischemic Stroke Using Imaging Analyses

**DOI:** 10.3390/diagnostics14121266

**Published:** 2024-06-15

**Authors:** Eun Hyun Cho, Kyung Hoon Park, Ji Hee Kim, Heejin Kim, Hyo-Jeong Lee, Jee Hye Wee

**Affiliations:** 1 Department of Otorhinolaryngology-Head & Neck Surgery, Hallym University Sacred Heart Hospital, Hallym University College of Medicine, Anyang 14068, Republic of Korea; ehc121@naver.com (E.H.C.); pzpdlh@hallym.or.kr (K.H.P.); heejin5020@daum.net (H.K.); hyobravo@gmail.com (H.-J.L.); 2 Department of Neurosurgery, Hallym University Sacred Heart Hospital, Hallym University College of Medicine, Anyang 14068, Republic of Korea; kimjihee.ns@gmail.com

**Keywords:** chronic rhinosinusitis, ischemic stroke, cerebrovascular disease, paranasal sinuses, anterior/posterior ethmoid

## Abstract

Recent studies have reported chronic rhinosinusitis (CRS) as an independent risk factor for stroke. However, the association with stroke depending on the affected sinuses has not been explored. This study aimed to elucidate the side- and sinus-specific relationship between CRS and ischemic stroke through imaging analyses. We retrospectively reviewed the medical records of patients who were diagnosed with ischemic stroke at a tertiary center. CRS was defined as having a total score of greater than or equal to 4, according to the Lund–Mackay scoring system, through brain magnetic resonance imaging or computed tomography. We investigated the side- and sinus-specific correlation between CRS and ischemic stroke. Subgroup analyses were performed for different age groups. CRS prevalence in patients with ischemic stroke was 18.4%, which was higher than the previously reported prevalence in the general population. Overall, there was no correlation between the directions of the CRS and ischemic stroke (*p* > 0.05). When each sinus was analyzed, the frontal (Cramer’s V = 0.479, *p* < 0.001), anterior (Cramer’s V = 0.396, *p* < 0.001)/posterior (Cramer’s V = 0.300, *p* = 0.008) ethmoid, and sphenoid (Cramer’s V = 0.383, *p* = 0.005) sinuses showed a statistically significant correlation with the side of stroke, but the maxillary sinus (Cramer’s V = 0.138, *p* = 0.208) did not. In subgroup analyses, a significant right-side correlation between the two diseases was observed in the older-age subgroup (≥65 years old, Cramer’s V = 0.142, *p* = 0.040). Diabetes mellitus (odds ratio = 1.596, 95% confidence interval = 1.204–2.116) was identified as an independent risk factor for having CRS in patients with ischemic stroke. CRS of the frontal, anterior/posterior ethmoid, and sphenoid sinuses has a directional relationship with ischemic stroke. Our results on which sinuses correlate with stroke advocate for the active surveillance of CRS in patients at high risk of ischemic stroke.

## 1. Introduction

Based on population-based survey responses or administrative database coding, the prevalence of chronic rhinosinusitis (CRS) is estimated to be 12.1% in the United States, 10.9% in Europe, 4.5% in Canada, and 6.95% in Korea [1,2]. It is particularly high in the elderly population, likely due to age-related changes in airway inflammation rather than the eosinophilic inflammatory response, which subsides with age [3]. CRS imposes a substantial socioeconomic burden on society [2]. The direct costs of CRS are estimated to be high, at USD 8.6 billion per year in the United States, and its indirect costs to society—the loss of quality of life and productivity associated with CRS—are estimated to cost an additional USD 12.8 billion [1]. Furthermore, CRS significantly impacts health-related quality of life in children as well as adults, affecting physical and mental health and social functioning, as well as placing emotional and time-related burdens on their families [4,5]. Stroke is a globally increasing disease as societies rapidly age, with 101 million cases reported worldwide in 2019. Stroke is the second leading cause of death worldwide, accounting for 11.6% of total deaths [6]. The burdens of CRS and stroke are major public health issues of growing importance.

CRS has recently been reported to be an independent risk factor for stroke [7,8,9]. In a cohort study using Taiwan’s national health insurance data, the acute and chronic rhinosinusitis groups had 1.39- and 1.34-fold higher risks of stroke, respectively, than the control group among 53,653 patients with rhinosinusitis matched with 214,624 controls by age and sex [7]. Another prospective cohort study in Taiwan revealed that patients with CRS were more likely to be diagnosed with ischemic stroke during a five-year follow-up period (hazard ratio (HR) = 1.34), although the difference in the risk of hemorrhagic stroke was not significant [8]. In another cohort study using national health insurance data in Korea, CRS was found to increase the risk of ischemic (HR = 1.76) and hemorrhagic (HR = 2.43) stroke [9]. The anatomical proximity of the paranasal sinuses to the brain and the potential spread of inflammation have been proposed as possible mechanisms that explain the association between CRS and stroke [7,8,9,10,11].

Most existing studies rely on diagnostic codes and lack in-depth research on CRS severity and disease status, the structural relationship between CRS and stroke, and how they contribute to stroke risk. Except for a small number of case reports, studies that have performed radiological analyses to reveal the anatomical relationship between the two diseases are rare. In a study of 173 patients who underwent brain magnetic resonance imaging (MRI) in Canada, incidental paranasal sinusitis was found in 11.6% of cases, and a significant association with cerebrovascular disease was observed, with an adjusted odds ratio (OR) of 5.2 [12]. However, the sample size was small, with only 38 stroke patients included, and comparative analysis of the affected sinuses or differences according to location was absent. In Poland, among 163 patients who underwent computed tomography (CT) and mechanical thrombectomy for ischemic stroke, CRS was observed in 36.8% of the patients [13]. Moderate-to-severe inflammatory sinus lesions were more frequently observed in patients with ischemic stroke, primarily involving the ethmoid sinuses, compared to a control group of 75 patients who underwent CT for non-vascular neurological disorders. However, this study also had limitations: a small sample size, restriction to patients who underwent mechanical thrombectomy, and a lack of analysis of anatomical locations.

We hypothesized that sinuses that are structurally adjacent to the brain parenchyma and major cerebral vessels, particularly the sphenoid and posterior ethmoid sinuses, might exert a direct influence on the brain and the occurrence of stroke. To validate the proposition derived from previous studies that the association between CRS and ischemic stroke is rooted in inflammation, we conducted a comparative analysis of the locations of CRS and stroke. This included a precise evaluation of the presence and severity of CRS using radiological examinations. This study primarily aimed to determine CRS prevalence in patients with ischemic stroke and investigate whether a spatial correlation existed between the location of the stroke and the affected sinuses, which would indicate a direct relationship between the two diseases. Secondly, this study aimed to identify factors that may contribute to the association between CRS and ischemic stroke. Our findings may provide valuable insights into treating patients with CRS and potentially preventing future stroke.

## 2. Materials and Methods

### 2.1. Study Populations

**Figure 1:**
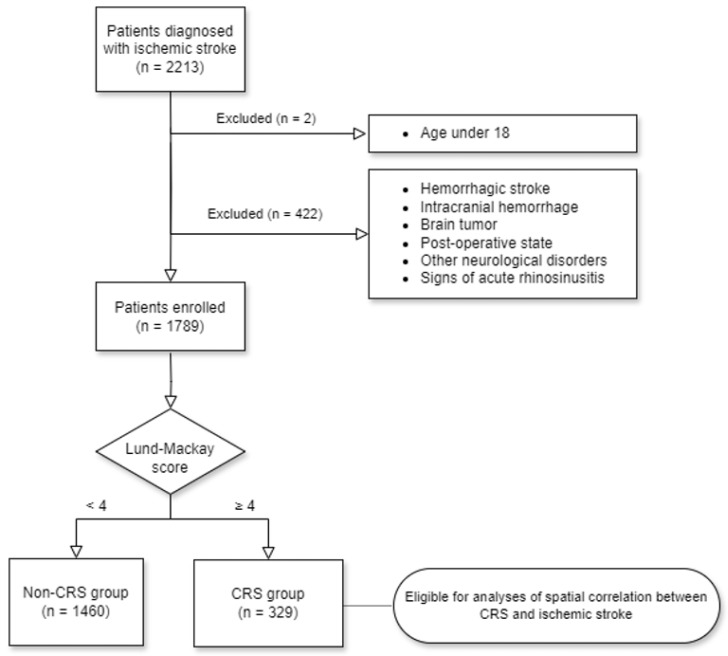
A flow chart of the participant selection process that was used in the present study.

We retrospectively reviewed the medical records of patients diagnosed with ischemic stroke via brain MRI or CT scanning at Hallym University Sacred Heart Hospital between January 2019 and May 2021 (*n* = 2213). The first time that we accessed the data was on 13 August 2021. To ensure the privacy and confidentiality of the participants, none of the authors had access to any information that could potentially reveal their identity during or after data collection. We excluded: patients diagnosed with intracranial hemorrhage or hemorrhagic stroke; brain tumors, including meningioma, metastasis, lymphoma, acoustic schwannoma, and multiple sclerosis; and other neurological disorders, such as Moyamoya disease and cerebral vascular malformations. Additionally, patients who were in a postoperative state following procedures such as craniectomy, craniotomy, thrombectomy, coil embolization, and surgical clipping of the cerebral aneurysm were excluded. Furthermore, patients aged under 18 years and those with signs of acute sinusitis (sinus air-fluid levels in imaging tests) were excluded (Figure 1).

All available imaging studies were reviewed to assess the presence of sinusitis and the side and location of the affected sinuses. Two independent examiners, who were blinded to the study, evaluated the brain images to determine the presence of mucosal thickening and opacification of each sinus using the Lund-Mackay (LM) scoring system. Although CT is the primary imaging modality for CRS severity staging, a robust correlation has been previously shown between LM scores obtained from CT and MRI scans, suggesting that MRI can serve as a reliable staging tool, using the same staging system as CT [14,15].

The Institutional Review Board of Hallym University (No. 2021-06-020) approved this study protocol. Written informed consent was waived due to the retrospective study design. All analyses adhered to the guidelines and regulations of the ethics committee of Hallym University.

### 2.2. Definition of CRS

An LM score of ≥4 out of 24 is defined as evidence of CRS, using brain MRI or CT scans [16]. We defined the side with the highest score as being dominant when the difference in LM scores between the right and left sides was >2. The presence of sinusitis in each sinus was defined as having a minimum LM score of 1 for that specific sinus in the CRS group, with a total LM score of ≥4.

### 2.3. Covariates

Patients’ age, sex, underlying diseases, and history of smoking or drinking were recorded. The underlying preexisting conditions known before the stroke diagnosis and newly identified conditions during hospitalization for stroke treatment were investigated. The chronic lung disease category mainly included bronchial asthma and chronic obstructive pulmonary disease (COPD), while arrhythmias included atrial fibrillation and atrial flutter. We divided the participants into those aged ≥ 65 years and those aged < 65 years for the subgroup analyses in the CRS group.

### 2.4. Statistical Analyses

The chi-square test and Cramer’s V were used to analyze the side correlation between ischemic stroke and CRS and to measure the strength of the association, respectively. Logistic regression analysis was used to assess the difference in side concordance according to CRS severity, as represented by the LM score. A multiple logistic regression model was used to identify the relevant factors contributing to the association between CRS and ischemic stroke. Any *p*-values of less than 0.05 were considered to indicate statistical significance. All statistical analyses were performed using SPSS 26.0 (IBM, Armonk, NY, USA).

## 3. Results

Among the 1789 patients included, 1049 men (58.6%) and 740 women (41.4%) were present whose mean age was 69.8 ± 13.8 (range, 18–99). Table 1 describes the baseline characteristics of the study participants. Significant differences were observed between the CRS and non-CRS patient groups in terms of sex (*p* < 0.001) and stroke side (*p* = 0.020). Various underlying conditions, such as hypertension (*p* = 0.887), dyslipidemia (*p* = 0.404), myocardial infarction (*p* = 0.054), arrhythmia (*p* = 0.188), chronic kidney disease (*p* = 0.179), and liver cirrhosis (*p* = 0.572), showed no significant differences in prevalence between the CRS group and the non-CRS group, with the exception of diabetes mellitus (*p* = 0.005). The proportion of patients with a history of alcohol consumption was significantly higher among those with CRS compared to those without (*p* = 0.001), but there was no significant difference in smoking history (*p* = 0.057).

### 3.1. CRS Prevalence in Patients with Ischemic Stroke

Among the 1789 patients, 329 were diagnosed with CRS, showing an 18.4% prevalence. The mean LM score of the patients diagnosed with CRS was 5.5 ± 2.5 (range: 4–18). A total of 33 cases (10.0%) of fungal sinusitis and 13 cases (4.0%) of nasal polyposis were diagnosed upon imaging. Among the 329 patients diagnosed with CRS, the frontal sinus, ethmoid sinus, maxillary sinus, and sphenoid sinus were involved in 84 (25.5%), 293 (89.1%), 322 (97.9%), and 115 (35.0%) patients, respectively. Additionally, 111 (33.7%) patients had right-sided strokes, 120 (36.5%) had left-sided strokes, and 98 (29.8%) had bilateral strokes.

### 3.2. Association between CRS and Ischemic Stroke with Respect to Side Correlation

The dominant side of the CRS and the side of the stroke did not show a correlation. When we analyzed the presence of ischemic stroke on the same side in relation to CRS, no correlation was observed (Table 2). However, when each sinus was analyzed, we observed a significant correlation between the stroke side and inflammatory lesion location in certain sinuses. Notably, a significant correlation was observed between the stroke side and dominant side of sinusitis in the frontal (*p* < 0.001, Cramer’s V = 0.479), anterior ethmoid (*p* < 0.001, Cramer’s V = 0.396), posterior ethmoid (*p* = 0.008, Cramer’s V = 0.300), and sphenoid (*p* = 0.005, Cramer’s V = 0.383) sinuses (Table 3). In contrast, when ischemic stroke occurred bilaterally, no significant correlation was observed in any of the sinuses (all *p* > 0.05, Table 3).

Subgroup analysis based on age (Supplementary Table S1) revealed a significant correlation between right-sided stroke and CRS in patients aged ≥ 65 years (*n* = 211; *p* = 0.040, Cramer’s V = 0.142). However, the analysis of the left side did not reveal a statistically significant association (*p* = 0.103, Cramer’s V = 0.112). In patients aged < 65 years (*n* = 118), the correlation between the direction of CRS and stroke in all analyses was insignificant (all *p* > 0.05).

### 3.3. Factors Related to the Association between CRS and Ischemic Stroke

After adjusting for age, sex, smoking status, and alcohol consumption in multiple logistic regression analysis, patients with diabetes mellitus (OR = 1.596, 95% CI = 1.204–2.116) had a higher risk of developing CRS. However, there were no significant differences in other comorbidities, such as hypertension (OR = 0.947, 95% CI = 0.719–1.249), dyslipidemia (OR = 0.891, 95% CI = 0.610–1.301), angina (OR = 0.610, 95% CI = 0.283–1.316), myocardial infarction (OR = 1.629, 95% CI = 0.916–2.899), arrhythmia (OR = 0.774, 95% CI = 0.448–1.334), COPD (OR = 1.130, 95% CI = 0.567–2.252), chronic kidney disease (OR = 0.761, 95% CI = 0.374–1.545), liver cirrhosis (OR = 0.561, 95% CI = 0.065–4.812), and thyroid disease (OR = 1.309, 95% CI = 0.641–1.301), between CRS and non-CRS groups (Table 4).

## 4. Discussion

No directional agreement was found when we conducted a radiological analysis of the entire CRS group to assess the concordance of the affected sides of CRS and ischemic stroke. However, CRS in specific sinuses may be directly related to ischemic stroke, depending on the sinus type. A significant side-specific association was observed between CRS and ischemic stroke in the frontal, anterior/posterior ethmoid, and sphenoid sinuses. The frontal sinus demonstrated the strongest association (Cramer’s V = 0.479), followed by the sphenoid sinus (Cramer’s V = 0.383), both exhibiting a moderate level of association. The ethmoid sinuses exhibited a weak but statistically significant association. These findings indicate a direct relationship between CRS in the frontal, ethmoid, and sphenoid sinuses and ischemic stroke. A causal relationship is suggested because the imaging tests were conducted at the onset of the stroke, allowing for the inference that CRS preceded the stroke.

The correlation between ischemic stroke and CRS in specific sinuses (frontal, ethmoid, and sphenoid) may be attributed to their proximity to the brain parenchyma and intracranial vasculature [13,17,18,19,20]. The sphenoid and posterior ethmoid sinuses are located near the internal carotid artery (ICA), separated by a thin 0.1-millimeter bony wall; in 8% of patients with CRS, the ICA bulges into the sinus [9]. Direct contact between the artery and sinus mucosa may be present in cases of bony dehiscence [10]. The paranasal sinuses, except the maxillary sinus, are delineated from the cranial cavity by a thin bony wall [10], allowing the direct invasion of sinusitis-associated infections into the brain. This potentially leads to well-recognized severe complications such as cranial nerve paralysis, brain abscesses, subdural or epidural empyema, and meningitis [7,8,12]. Intracranial infections can cause cerebral vasculitis by extending to the intracranial arteries, impairing blood flow to the brain and inducing cerebral ischemia [11]. Non-infectious vasculopathy caused by CRS is also believed to contribute to ischemic stroke development. In patients with CRS, localized concentrations of inflammatory cytokines within the sinus fluid and mucosa have been observed [8,10]. Chronic sinus inflammation can compromise endothelial cell integrity because inflammatory cytokines such as interleukin (IL)-1, IL-6, and C-reactive protein activate the immune cells and smooth muscle cells in the subendothelial layer. This process accelerates atherogenesis, culminating in atherosclerosis and the subsequent ischemic stroke risk [7,8,11]. Moreover, inflammatory cytokines can activate the coagulation cascade, increasing thrombus formation and the likelihood of thromboembolic events [8,11].

Invasive fungal sinusitis, characterized by its rapid progression and high mortality rate, has occasionally been reported to cause ischemic stroke when it extends from the sphenoid sinuses to the skull base and the brain, affecting major vessels of the brain such as the basilar and carotid arteries [17,18,21]. Apart from invasive fungal sinusitis, there have been a lot of case reports suggesting a correlation between sphenoid sinusitis and ischemic stroke. Wong et al. reported on several pediatric patients in whom sphenoid sinus inflammation extended to the cavernous ICA segment, which was near the sphenoid sinus [20]. Barreto et al. observed sphenoid sinusitis in four patients who experienced ischemic stroke due to stenosis or occlusion of the ipsilateral ICA, as seen on MRI scans of patients spanning from children to the elderly [10]. A case report of acute sphenoid sinusitis complicated by ischemic stroke was previously reported, with stenosis of the left ICA and anterior and middle cerebral arteries detected on the MRI [22]. In another case report, a patient diagnosed with left superior ophthalmic vein thrombosis due to chronic sphenoid sinusitis later developed an ischemic infarction of the terminal branch of the supraclinoid segment of the left ICA [19]. Furthermore, an association between ischemic stroke and, predominantly, the ethmoid sinus was previously found, suggesting that the ethmoid sinus, which is characterized by a complex network of ethmoid cells lined with mucosa and accompanied by anterior and posterior ethmoid arteries, may facilitate the transport of inflammatory mediators generated within the sinuses [13]. Although rare, these case reports collectively suggest that local inflammation of the sinuses plays a significant role in triggering acute cerebral ischemia [7,13]. No association was found between the CRS of the maxillary sinus and stroke in terms of direction, which may be due to its being relatively distant from the brain and major cerebral vessels compared to other sinuses. Furthermore, obtaining significant results may be challenging given that nearly all the patients (specifically 322 patients) diagnosed with CRS had partial haziness in at least one of the bilateral maxillary sinuses.

CRS prevalence in patients with ischemic stroke in our study was 18.4%, which was higher than the previously reported prevalence in the general population (US, 12.1%; Europe, 10.9%; Canada, 4.5%; Korea, 6.95%) [1,2,23]. Large-scale studies aimed at establishing CRS epidemiology and prevalence rely mostly on questionnaire-based evaluations. However, concerns have been raised regarding the limitations of this methodology because CRS diagnosis should be based on a combined assessment of subjective sinonasal symptoms and objective evidence of tissue inflammation via sinus radiology or nasal endoscopy [1,23]. Diagnosing CRS based on symptoms alone can be sensitive but also prone to a high false-positive rate because CRS symptoms can overlap with those of other prevalent conditions, such as allergic or non-allergic rhinitis and acute rhinosinusitis [1,2,23]. A significant proportion of individuals with self-reported symptoms did not exhibit radiological evidence or were diagnosed by physicians as not having CRS, as reported by several previous studies. This discrepancy raises concerns about overestimating CRS prevalence in survey-based studies [1,23]. Furthermore, sinus CT scans have previously shown good sensitivity and above-average specificity for diagnosing CRS, and an LM score of ≥4 is more likely to indicate true CRS [16]. Given that our study diagnosed CRS using objective evidence of tissue inflammation, the overestimation of its prevalence is less concerning. This enables us to assert that CRS prevalence in the ischemic stroke patient population calculated in our study is indeed higher than that in the general population, as prevalence in the general population is derived from questionnaire-based studies. The higher CRS prevalence in patients with ischemic stroke indicates a potential association between these two conditions.

Subgroup analysis according to age revealed a significant association between the presence of right-side CRS and right-side stroke in the CRS group aged ≥ 65. Although the association on the left side was insignificant, considering the absence of any association in patients aged < 65, the association between ischemic stroke and CRS can be inferred to be stronger in an elderly population aged ≥ 65. The discrepancy observed between adult and elderly patients can be attributed to the recently proposed differences in the CRS histopathological characteristics between these two groups [24,25,26]. Elderly CRS patients exhibit a distinct endotype characterized by a more pro-inflammatory and neutrophilic immune response, along with elevated IL-1β, IL-6, IL-8, and tumor necrosis factor (TNF)-α levels in the mucus, regardless of the polyp status [25]. In contrast to adult patients with CRS, in whom TH_2_-skewed responses with eosinophilia are believed to play a critical role in disease development, microbiome dysbiosis and epithelial barrier dysfunction may influence CRS pathogenesis in the elderly [26]. Renteria et al. explained age-related changes in three elements of CRS pathogenesis: the epithelial barrier, host immunity, and the microbiome [26]. Aging causes decreased mucociliary clearance and thinner epithelium and basal cell layers, as well as reduced S100 protein levels, which are crucial for epithelial barrier function [26,27]. Age-related increase in inflammation, known as “inflammaging,” contributes to the complexity of the dysbiotic sinus microbiome in the elderly; the dysfunction of innate and adaptive immune mechanisms associated with aging, known as “immunosenescence,” may hinder bacterial clearance and increase susceptibility to infection [26]. Elderly patients with CRS exhibit a decline in eosinophil function, accompanied by a tendency toward increased systemic inflammation [24,26]. Thus, CRS in the elderly can be assumed to have a distinct pathophysiology that may contribute to the mechanisms involved in inducing ischemic stroke, such as perivascular inflammation, atherosclerotic initiation, thrombosis, and vascular spasms induced by exposure to inflammatory mediators [7,8,11,17].

Several large-scale studies have reported a higher prevalence of comorbidities in patients with CRS than in controls. Two nationwide population-based studies in Taiwan reported that patients with CRS were more prone to having comorbidities such as coronary heart disease, diabetes, hypertension, and dyslipidemia than those in a comparison cohort [6,8]. A population-based study conducted in Korea reported a higher prevalence of stroke, ischemic heart disease, migraine, chronic kidney disease, depression, sleep disorder, and COPD among patients with CRS [9]. Previous studies have revealed diverse comorbidities associated with CRS, including asthma, cardiovascular conditions such as acute myocardial infarction, and depression [28]. However, in the present study, there were no significant differences in the prevalence of comorbidities between the CRS and non-CRS groups, except for diabetes. This can be explained by the fact that this study only included patients with ischemic stroke, and the results suggest that in the CRS group, the association with ischemic stroke may be direct, rather than this being due to the effect of other underlying diseases. Further studies including a control group of patients without ischemic stroke should be conducted to confirm this causal relationship.

The present study has several limitations. First, we were unable to meet the CRS diagnostic criteria requiring at least 12 weeks of follow-up because enrolling only those patients who underwent imaging follow-up at intervals of over 12 weeks would result in the exclusion of a considerable number of individuals. However, we excluded patients who exhibited air-fluid levels in the sinuses from imaging studies to exclude cases of acute rhinosinusitis. Second, we lacked subjective data regarding sinonasal symptoms and scores and the history of treatment following the EPOS 2020 guidelines [29]. We also did not analyze whether conservative or surgical treatments for CRS affected the course of ischemic stroke, due to the retrospective nature of our study and limited documentation on whether or not patients received treatment for CRS. Future studies should investigate whether different CRS treatments influence the risk or progression of ischemic stroke. Third, we did not specify the vessel responsible for causing the stroke. Subsequent research should analyze the location of the causative vessel and the affected sinus to uncover a more robust relationship. Additionally, the study period coincided with the COVID-19 pandemic, potentially biasing our results. However, the present study included patients both before (until 29 February 2020) and during COVID-19 (from 1 March 2020) [30], with the prevalence of CRS being 21.2% (181/853) before COVID-19 and 15.8% (148/936) during COVID-19, so the impact is expected to be minimal. Potential sources of bias in the evaluation of CRS include interobserver variability (minimized by employing two independent otolaryngology specialists) and the tendency of MRI grading to overestimate mucosal thickening and sinusitis. Furthermore, as we did not include a control group without ischemic stroke, a causal relationship between CRS and ischemic stroke could not be determined. Although we can infer that CRS preceded ischemic stroke because CRS presence was evaluated based on imaging tests conducted at the onset of ischemic stroke, it is noteworthy that the present study was cross-sectional, which does not allow us to establish a cause-and-effect relationship. Further studies, including a control group of patients without stroke, should be conducted to confirm a causal relationship.

## 5. Conclusions

The directional concordance between CRS and ischemic stroke suggests a direct association between the two conditions, supporting the possibility of a causal relationship. These results can serve as evidence to recommend close monitoring or active treatment for CRS in patients with a history of stroke or in those at high risk of stroke. Future studies should include a control group and should conduct imaging analyses to elucidate a causal relationship between CRS and ischemic stroke. Furthermore, the impact and underlying mechanisms of CRS on the course of ischemic stroke, treatment outcomes, and prognosis (including poststroke disability) should be investigated. This will help determine the extent to which proactive CRS treatment can contribute to treating and preventing ischemic stroke.

## Figures and Tables

**Table 1:** Demographic characteristics of the participants.

Variables	Total (*n* = 1789)	CRS		
		Non-CRS (*n* = 1460)	CRS (*n* = 329)	*p*-Value
Age (years old, mean ± SD)	69.8 ± 13.8	69.9 ± 12.6	69.8 ± 14.0	0.805
Sex (*n*, %)				<0.001 ^1^
Male	1049 (58.6)	813 (55.7)	236 (71.7)	
Female	740 (41.4)	647 (44.3)	93 (28.3)	
Side of stroke (*n*, %)				0.020 ^1^
Right	690 (38.6)	579 (39.7)	111 (33.7)	
Left	667 (37.3)	547 (37.5)	120 (36.5)	
Both	432 (24.1)	334 (22.9)	98 (29.8)	
LM score (mean ± SD)	1.66 ± 2.33	0.78 ± 1.02	5.53 ± 2.53	<0.001 ^2^
Smoking status				0.057
never smoker	1359 (76.0)	1123 (82.0)	236 (76.1)	
ex-smoker	54 (3.0)	41 (3.0)	13 (4.2)	
current smoker	266 (14.9)	205 (15.0)	61 (19.7)	
missing	110 (6.1)			
Alcohol consumption (*n*, %)				0.001 ^1^
<1 time a week	1227 (68.6)	1023 (74.0)	359 (65.0)	
≥1 time a week	469 (26.2)	204 (26.0)	110 (35.0)	
missing	110 (6.1)			
Hypertension (*n*, %)	1016 (56.8)	828 (56.7)	188 (57.1)	0.887
Diabetes mellitus (*n*, %)	512 (28.6)	397 (27.2)	115 (35.0)	0.005 ^1^
Dyslipidemia (*n*, %)	260 (14.5)	217 (14.9)	43 (13.1)	0.404
Angina (*n*, %)	68 (3.8)	59 (4.0)	9 (2.7)	0.263
Myocardial infarction (*n*, %)	70 (3.9)	51 (3.5)	19 (5.8)	0.054
Arrhythmia (*n*, %)	122 (6.8)	105 (7.2)	17 (5.2)	0.188
Chronic lung disease (*n*, %)	59 (3.3)	46 (3.2)	13 (4.0)	0.463
Chronic kidney disease (*n*, %)	79 (4.4)	69 (4.7)	10 (3.0)	0.179
Liver cirrhosis (*n*, %)	9 (0.5)	8 (0.5)	1 (0.3)	0.572
Thyroid disease (*n*, %)	65 (3.6)	54 (3.7)	11 (3.3)	0.756

^1^ Chi-square test or Fischer’s exact test, significance at *p* < 0.05. ^2^ Mann–Whitney U test, significance at *p* < 0.05. CRS = chronic rhinosinusitis, SD = standard deviation, LM score = Lund–Mackay score.

**Table 2:** The side correlation between chronic rhinosinusitis and ischemic stroke.

		**Side of stroke,** *n* **(%)**		**Total**	***p*** **-value**	**Cramer’s V**
		**Both**	**Unilateral**			
Side of CRS	Both	74 (30.3)	170 (69.7)	244 (74.2)	0.716	0.020
	Unilateral	24 (28.2)	61 (71.8)	85 (25.8)		
Total		98 (29.8)	231 (70.2)	329		
		**Side of stroke,** *n* **(%)**		**Total**	***p*** **-value**	**Cramer’s V**
		**Rt.**	**Lt.**			
Dominant side of CRS	Rt.	19 (61.3)	12 (38.7)	31 (50.8)	0.054	0.246
	Lt.	11 (36.7)	19 (63.3)	30 (49.2)		
Total		30 (49.2)	31 (50.8)	61		
		**Presence of Rt. stroke,** *n* **(%)**		**Total**	***p*** **-value**	**Cramer’s V**
		**Yes**	**No**			
Presence of Rt. CRS	Yes	188 (65.1)	101 (34.9)	289 (87.8)	0.122	0.085
	No	21 (52.5)	19 (47.5)	40 (12.2)		
Total		209 (63.5)	120 (36.5)	329		
		**Presence of Lt. stroke,** *n* **(%)**		**Total**	***p*** **-value**	**Cramer’s V**
		**Yes**	**No**			
Presence of Lt. CRS	Yes	192 (67.6)	92 (32.4)	284 (86.3)	0.195	0.071
	No	26 (57.8)	19 (42.2)	45 (13.7)		
Total		218 (66.3)	111 (33.7)	329		

CRS = chronic rhinosinusitis.

**Table 3:** The side correlation between chronic rhinosinusitis and ischemic stroke for each sinus.

		**Side of stroke,** *n* **(%)**		**Total**	***p*** **-value**	**Cramer’s V**
		**Both**	**Unilateral**			
Side of frontal sinusitis	Both	4 (23.5)	13 (76.5)	17 (20.2)	0.705	0.041
	Unilateral	13 (19.4)	54 (80.6)	67 (79.8)		
Total		17 (20.2)	67 (79.8)	84		
		**Side of stroke,** *n* **(%)**		**Total**	***p*** **-value**	**Cramer’s V**
		**Rt.**	**Lt.**			
Dominant side of frontal sinusitis	Rt.	22 (75.9)	7 (24.1)	29 (53.7)	<0.001 ^1^	0.479
	Lt.	7 (28.0)	18 (72.0)	25 (46.3)		
Total		29 (53.7)	25 (46.3)	54		
		**Side of stroke,** *n* **(%)**		**Total**	***p*** **-value**	**Cramer’s V**
		**Both**	**Unilateral**			
Side of ant. ethmoid sinusitis	Both	41 (29.5)	98 (70.5)	139 (54.9)	0.803	0.016
	Unilateral	32 (28.1)	82 (71.9)	114 (45.1)		
Total		73 (28.9)	180 (71.1)	253		
		**Side of stroke,** *n* **(%)**		**Total**	***p*** **-value**	**Cramer’s V**
		**Rt.**	**Lt.**			
Dominant side of ant. ethmoid sinusitis	Rt.	25 (61.0)	16 (39.0)	41 (50.0)	<0.001 ^1^	0.396
	Lt.	9 (22.0)	32 (78.0)	41 (50.0)		
Total		34 (41.5)	48 (58.5)	82		
		**Side of stroke,** *n* **(%)**		**Total**	***p*** **-value**	**Cramer’s V**
		**Both**	**Unilateral**			
Side of post. ethmoid sinusitis	Both	17 (27.0)	46 (73.0)	63 (37.3)	0.958	0.004
	Unilateral	29 (27.4)	77 (72.6)	106 (62.7)		
Total		46 (27.2)	123 (72.8)	169		
		**Side of stroke,** *n* **(%)**		**Total**	***p*** **-value**	**Cramer’s V**
		**Rt.**	**Lt.**			
Dominant side of post. ethmoid sinusitis	Rt.	28 (63.6)	16 (36.4)	44 (57.1)	0.008 ^1^	0.300
	Lt.	11 (33.3)	22 (66.7)	33 (32.9)		
Total		39 (50.6)	38 (49.4)	77		
		**Side of stroke,** *n* **(%)**		**Total**	***p*** **-value**	**Cramer’s V**
		**Both**	**Unilateral**			
Side of sphenoid sinusitis	Both	16 (43.2)	21 (56.8)	37 (32.2)	0.145	0.136
	Unilateral	23 (29.5)	55(70.5)	78 (67.8)		
Total		39 (33.9)	76 (66.1)	115		
		**Side of stroke,** *n* **(%)**		**Total**	***p*** **-value**	**Cramer’s V**
		**Rt.**	**Lt.**			
Dominant side of sphenoid sinusitis	Rt.	21 (67.7)	10 (32.3)	31 (56.4)	0.005 ^1^	0.383
	Lt.	7 (29.2)	17 (70.8)	24 (43.6)		
Total		28 (50.9)	27 (49.1)	55		
		**Side of stroke,** *n* **(%)**		**Total**	***p*** **-value**	**Cramer’s V**
		**Both**	**Unilateral**			
Side of maxillary sinusitis	Both	57 (28.5)	143 (71.5)	200 (62.1)	0.509	0.037
	Unilateral	39 (32.0)	83 (68.0)	122 (37.9)		
Total		96 (29.8)	226 (70.2)	322		
		**Side of stroke,** *n* **(%)**		**Total**	***p*** **-value**	**Cramer’s V**
		**Rt.**	**Lt.**			
Dominant side of maxillary sinusitis	Rt.	23 (52.3)	21 (47.7)	44 (53.0)	0.208	0.138
	Lt.	15 (38.5)	24 (61.5)	39 (47.0)		
Total		38 (45.8)	45 (54.2)	83		

^1^ *p* < 0.05 by χ^2^ test.

**Table 4:** Odds ratios (95% confidence interval) for chronic rhinosinusitis in ischemic stroke patients, shown according to underlying diseases.

	Crude	*p*-Value	Adjusted	*p*-Value
Hypertension		0.887		0.701
Yes	1.018 (0.779–1.296)		0.947 (0.719–1.249)	
No	1.000		1.000	
Diabetes mellitus		0.005		0.020 ^1^
Yes	1.439 (1.116–1.855)		1.596 (1.204–2.116)	
No	1.000		1.000	
Dyslipidemia		0.405		0.551
Yes	0.861 (0.606–1.224)		0.891 (0.610–1.301)	
No	1.000		1.000	
Angina		0.266		0.208
Yes	0.668 (0.328–1.361)		0.610 (0.283–1.316)	
No	1.000		1.000	
Myocardial infarction		0.056		0.097
Yes	1.693 (0.986–2.908)		1.629 (0.916–2.899)	
No	1.000		1.000	
Arrhythmia		0.190		0.356
Yes	0.703 (0.415–1.191)		0.774 (0.448–1.334)	
No	1.00		1.000	
Chronic lung disease		0.464		0.728
Yes	1.265 (0.675–2.369)		1.130 (0.567–2.252)	
No	1.000		1.000	
Chronic kidney disease		0.182		0.449
Yes	0.632 (0.322–1.240)		0.761 (0.374–1.545)	
No	1.000		1.000	
Liver cirrhosis		0.578		0.598
Yes	0.553 (0.069–4.440)		0.561 (0.065–4.812)	
No	1.000		1.000	
Thyroid disease		0.756		0.460
Yes	0.901 (0.466–1.742)		1.309 (0.641–1.301)	
No	1.000		1.000	

^1^ Logistic regression was performed with adjustments for age, sex, smoking status, and alcohol consumption, considering *p* < 0.05 as significant.

## Data Availability

Data are contained within the article and supplementary materials.

## Supplementary Materials

The following supporting information can be downloaded at: https://www.mdpi.com/article/10.3390/diagnostics14121266/s1, Table S1: The side correlation between chronic rhinosinusitis and ischemic stroke, according to age subgroup.
