# Supplementary material for: Side- and Sinus-Specific Relationships between Chronic Rhinosinusitis and Ischemic Stroke Using Imaging Analyses
Source: Diagnostics (Basel). 2024 Jun 15;14(12):1266. doi: 10.3390/diagnostics14121266 (PMC11203191; doi:10.3390/diagnostics14121266)
Supplement: Supplementary file 1 [file diagnostics-14-01266-s001.zip › diagnostics-3005686-supplementary.pdf]

Table S1. The side correlation between the chronic rhinosinusitis and ischemic stroke according to age subgroup.

**Age ≥ 65 years**		**Side of stroke, n (%)**		**Total**	**P-value**	**Cramer's V**
		**Both**	**Unilateral**			
Side of CRS	Both	46 (30.9)	103 (69.1)	149 (70.6)		
	Unilateral	16 (25.8)	46 (74.1)	62 (29.4)	0.462	0.051
Total		62 (29.4)	149 (70.6)	211		

**Age < 65 years**		**Side of stroke, n (%)**		**Total**	**P-value**	**Cramer's V**
		**Both**	**Unilateral**			
Side of CRS	Both	28 (29.5)	67 (70.5)	95 (80.5)		
	Unilateral	8 (34.8)	15 (65.2)	23 (19.5)	0.620	0.046
Total		36 (30.5)	82 (69.5)	118		

**Age ≥ 65 years**		**Side of stroke, n (%)**		**Total**	**P-value**	**Cramer's V**
		**Rt.**	**Lt.**			
Dominant side of CRS	Rt.	14 (70.0)	6 (30.0)	20 (43.5)		
	Lt.	9 (34.6)	17 (65.4)	26 (56.5)	0.310	0.030
Total		23 (50.0)	23 (50.0)	46		

**Age < 65 years**		**Side of stroke, n (%)**		**Total**	**P-value**	**Cramer's V**
		**Rt.**	**Lt.**			
Dominant side of CRS	Rt.	5 (50.0)	5 (50.0)	10 (66.7)		
	Lt.	3 (60.0)	2 (40.0)	5 (33.3)	0.714	0.094
Total		8 (53.3)	4 (46.7)	15		

**Age ≥ 65 years**		**Presence of Rt. stroke, n (%)**		**Total**	**P-value**	**Cramer's V**
		**Yes**	**No**			
Presence of Rt. CRS	Yes	118 (65.9)	61 (34.1)	179 (84.8)		
	No	15 (46.9)	17 (53.1)	32 (15.2)	0.040 [1]	0.142
Total		133 (63.0)	78 (37.0)	211		

**Age < 65 years**		**Presence of Rt. stroke, n (%)**		**Total**	**P-value**	**Cramer's V**
		**Yes**	**No**			
Presence of Rt. CRS	Yes	70 (63.6)	40 (36.4)	110 (93.2)		
	No	6 (75.0)	2 (25.0)	8 (6.8)	0.517	0.060
Total		76 (64.4)	42 (35.6)	118		

**Age ≥ 65 years**		**Presence of Lt. stroke, n (%)**		**Total**	**P-value**	**Cramer's V**
		**Yes**	**No**			
Presence of Lt. CRS	Yes	124 (65.8)	57 (31.5)	181 (85.8)		
	No	16 (53.3)	14 (46.7)	30 (14.2)	0.103	0.112
Total		140 (66.4)	71 (33.6)	211		

**Age < 65 years**		**Presence of Lt. stroke, n (%)**		**Total**	**P-value**	**Cramer's V**
		**Yes**	**No**			
Presence of Lt. CRS	Yes	68 (66.0)	35 (34.0)	103 (87.3)		
	No	10 (66.7)	5 (33.3)	15 (12.7)	0.961	0.005
Total		78 (66.1)	40 (33.9)	118		

[1] $P < 0.05$ by $\chi 2$ test
